# Professional identity, job satisfaction, and turnover intention among Chinese novice nurses: A cross-sectional study

**DOI:** 10.1097/MD.0000000000036903

**Published:** 2024-01-19

**Authors:** Ying Zhong, Huan Ma, Cui-Cui Zhang, Qin-Ying Jiang, Jun Li, Chang-Ju Liao, Yu-Fen Liang, Li Shu

**Affiliations:** a Nursing Department, Zigong First People’s Hospital, Sichuan, China; b Zigong Academy of Medical Sciences, Sichuan, China; c School of Nursing, Sichuan Vocational College of Health and Rehabilitation, Sichuan, China

**Keywords:** China, job satisfaction, novice nurses, professional identity, turnover intention

## Abstract

The world is faced with challenges due to a growing aging population and the increasing burden of chronic disease. The acute shortage of nurses and high turnover rates, particularly among novice nurses, are of great concern in many countries. Several studies have shown that turnover intention among nurses is influenced by professional identity and job satisfaction. However, to the best of our knowledge, no studies have examined this issue in the context of novice nurses. Thus, the present study aimed to explore the relationship between professional identity, job satisfaction, and turnover intention among novice nurses in China. From March 18 to April 23, 2022, a cross-sectional survey was carried out involving 532 novice nurses recruited from four public hospitals in Sichuan Province, China. Among the sample, 526 questionnaires were retrieved, with an effective response rate of 98.87%. The mean scores for turnover intention, professional identity, and job satisfaction were 13.02 ± 3.94, 36.17 ± 7.98, and 111.02 ± 21.46, respectively. High turnover intention was observed among novice nurses, of whom 54.37% (286/526) had high or very high turnover intention. Professional identity and job satisfaction among novice nurses were moderate. In terms of demographic characteristics, “Whether living with relatives” and “Monthly income” had a statistically significant impact on the turnover intention of novice nurses (*P <* .05). Both professional identity (*r* = −0.459) and job satisfaction (*r* = −0.517) were significantly and moderately negatively correlated with turnover intention (*P* < .01). The results of the multivariate linear regression analysis revealed that variables including “Whether living with relatives,” “Professional identity,” “Control and responsibility for work,” and “Benefits” jointly accounted for 29.9% of the variance related to turnover intention among novice nurses. “Whether living with relatives,” “Professional identity,” “Control and responsibility for work,” and “Benefits” were highly predictive of turnover intention levels among novice nurses. Hence, potential predictors of turnover intention should be considered, and intervention research should be conducted to reduce the level of turnover intention among novice nurses.

## 1. Introduction

The world is facing various challenges in response to a growing aging population and increasing burden of chronic disease. A forecast has predicted that by 2030, 25% of Europe’s population and 20% of America’s population will be over 65 years old^[1]^ while 26.19% of China’s population will be aged 60 and older.^[2]^ About 21% of the elderly aged ≥ 60 years in India have at least one chronic disease,^[3]^ the latter of which affects up to 81.1% of older people aged ≥ 60 years in China,^[4]^ which has dramatically increased the demand for nursing resources. Moreover, the COVID-19 pandemic has intensified nursing staff workloads.^[5]^ Nowadays, the acute shortage of nurses and high turnover rates, including among novice nurses, are of great concern in many countries.

High turnover rates among nurses place a significant financial burden on the healthcare system.^[6,7]^ The financial burden of the total nursing turnover cost in the United States was estimated to be $5908,294,^[8]^ ranging from $10,098–$88,000 per employee.^[8,9]^ Moreover, this can reduce healthcare accessibility, decrease patient safety, compromise the quality of patient care, and affect patient outcomes.^[6,7,10–12]^According to research findings, turnover intention is a critical precursor of turnover among nurses.^[6,13]^ Turnover intention mirrors staff members’ views of the organization. Turnover intention among nurses essentially means that nurses have a conscious intention to vacate their job position in the period ahead.^[14,15]^ Several international studies examining turnover intention among nurses have reported that the turnover intention among this group was high, ranging from 23.01% to 48.6%.^[5,6,16–22]^ Related research found that younger nurses were more likely to have higher turnover intention,^[5,16]^ as more than 33.3% of novice nurses had left their first employment position.^[23]^ Novice nurses are also regarded as newly registered graduates with 3 years of work experience.^[24]^ Novice nurses have a high turnover intention, which results in a higher turnover rate and a greater shortage of nurses, creating a vicious circle.

Professional identity is an abstract concept composed of the individual values, attitudes, and beliefs associated with one’s profession.^[25–28]^ But a professional identity is never written in stone; it can be shaped by experiences and expectations, which are further refined as novice nurses transition and integrate into the workforce. Nurses’ professional identities can also be shaped by their experience and expectations.^[25,27,28]^ In addition, the construction of an individual’s professional identity is influenced by their public image, work environment, and work values.^[25]^ Sun et al ^[29]^ found a moderate negative relationship between the professional identity of novice nurses and burnout, whereas professional identity was highly positively related to quality of work life.

Job satisfaction is typically defined as a pleasant and positive emotional state that is experienced as a result of an individual’s job environment and experiences,^[30–32]^ and it is regarded as the most important factor that contributes to the retention of novice nurses.^[33]^ Job satisfaction is usually characterized by eight factors: “Benefits,” “Scheduling,” “Balance between family and work,” “Relations with colleagues,” “Social opportunities,” “Opportunities for professional development,” “Praise and recognition of work,” and “Control and responsibility for work.”^[34]^ Previous research has found that job satisfaction among nurses is either moderate or low.^[35–37]^ Professional identity is positively related to job satisfaction among nursing staff and professional identity programs can enhance job satisfaction.^[14,38]^

A series of studies has shown that turnover intention is influenced by professional identity and job satisfaction.^[26,30,39,40]^ Related studies involving correlations between professional identity, job satisfaction, and turnover intention were conducted among health workers;^[14,26,41,42]^ however, to the best of our knowledge, no studies have examined this situation in the context of novice nurses. Thus, the present study aimed to explore the relationship between professional identity, job satisfaction, and turnover intention among novice nurses in China.

## 2. Materials and methods

### 2.1. Study design

This was a multicenter cross-sectional research.

### 2.2. Setting and recruiters

A questionnaire survey was conducted between March 18 and April 23, 2022. We obtained the consent of each hospital involved in the study. All participants were informed of the purpose of the study and provided their informed consent through a questionnaire survey. Convenience sampling was used to investigate novice nurses in four public hospitals in three cities in Sichuan Province, China. All data were pseudonymized. The selection criteria were as follows: (1) registered nurses; (2)≤3 working years; (3) clinical on-the-job nurses; and (4) agreed to voluntarily participate in this study. The exclusion criteria were as follows: (1) previous history of mental illness and (2) intern nurses and refresher nurses. A total of 532 novice nurses completed the questionnaires, of which 526 were retrieved, with an effective response rate of 98.87%.

### 2.3. Questionnaires

The following four measurement tools were employed: a socio-demographic characteristics questionnaire, turnover intention scale, professional identity scale, and McCoskey/Mueller Satisfaction Scale (MMSS).

#### 2.3.1. Socio-demographic Characteristics.

The socio-demographic characteristics questionnaire covered information related to gender, age, years of work experience, education level, marital status, monthly income (China Yuan, CNY), and whether the respondents were living with relatives (Table 1).

**Table 1:** Demographic characteristic of the novice nurses (N = 526).

Variables	N (%)	Turnover intention Mean ± SD
Gender		*P = .420*
Male	47 (8.94)	13.47 ± 3.89
Female	479 (91.06)	12.98 ± 3.95
Age (years)		*P = *.105
<20	15 (2.85)	11.40 ± 3.92
≥20	511 (97.15)	13.07 ± 3.94
Working years (years)		*P = .708*
<1	172 (32.70)	13.06 ± 3.67
1 to 2	192 (36.50)	13.17 ± 3.95
2 to 3	162 (30.80)	12.82 ± 4.22
Education level		*P = *.087
Associate degree and below	400 (76.05)	12.86 ± 3.95
Bachelor degree	119 (22.62)	13.43 ± 3.94
Master degree	7 (1.33)	15.57 ± 2.51
Marital status		*P = *.416
Unmarried	472 (89.73)	13.07 ± 3.90
Married	54 (10.27)	12.61 ± 4.33
Monthly income (CNY)		*P = *.011*
<4000	316 (60.08)	13.23 ± 3.99
4000–6000	158 (30.04)	13.12 ± 3.69
>6000	52 (9.89)	11.48 ± 4.12
Whether living with relatives		*P = *.000**
Yes	193 (36.69)	11.94 ± 3.84
No	333 (63.31)	13.65 ± 3.87

CNY = China Yuan, SD = standard deviation

* *P* < .05

** *P* < .01

#### 2.3.2. Turnover intention.

Novice nurses’ turnover intention was evaluated using the Turnover Intention Scale developed by Michaels^[43]^ which was adapted for use in the Chinese population by Li and Li^[44]^ The scale consists of 6 items and three dimensions and is measured according to a 4-point Likert scale ranging from 1 (never) to 4 (often). A total average score ≤ 1 indicates very low turnover intention, >1 and≤2 indicate low turnover intention, >2 and≤3 indicate high turnover intention, and>3 indicates very high turnover intention. The Cronbach’s α coefficient of the Chinese version was 0.873, and its content validity was 67.67%. In this study, the reliability of the Turnover Intention Scale was Cronbach’s α = 0.806.

#### 2.3.3. Professional identity.

Novice nurses’ professional identity was evaluated using the Chinese version of the Professional Identity Scale, which is composed of 10 items and scored on a 5-point Likert scale ranging from 1 (completely inconsistent) to 5 (fully compliant).^[45]^ The level of professional identity was categorized as follows:10 to 20 (disapproval), 21 to 40 (general identity), and 41 to 50 (high identity). The Cronbach’s α for the scale was 0.930. In this study, the Cronbach’s α for the Professional Identity Scale was 0.952.

#### 2.3.4. MMSS.

Novice nurses’ job satisfaction was evaluated using the MMSS revised by Mueller^[34]^. The scale consists of 31 items and eight dimensions, which are scored according to a 5-point Likert scale ranging from 1 (very dissatisfied) to 5 (very satisfied). Cronbach’s α coefficient of the scale was 0.950. In this study, the reliability of the MMSS was Cronbach’s α = 0.961.

### 2.4. Ethical approval

The study was approved by the Research Ethics Committee of the Zigong First People’s Hospital on March 15, 2022 (No. 20220515). All participants were informed of the purpose of the study and informed consent was obtained from the study participants through a questionnaire survey.

### 2.5. Data analysis

The statistical analysis was performed with IBM SPSS Statistics 26.0. The socio-demographic characteristics of the recruiters were expressed numerically in the format of the mean and standard deviation, and frequencies with percentages. The bivariate relationships of turnover intention, professional identity, and MMSS were captured by performing Pearson’s correlation analysis. Multivariate linear regression analysis was used to assess the effect of professional identity, MMSS, and “whether living with relatives” on turnover intention. The significance level was set at *P* < .05 (two-tailed).

### 2.6. Patient and public involvement

Patients or members of the public were not involved in the design, reporting, or dissemination plans of our research.

## 3. Results

### 3.1. Demographic characteristics of respondents

The demographic characteristics of the 526 participants are presented in Table 1. Most respondents were female (91.06%), had less than 2 years of work experience (69.20%), held an associate degree and below (76.05%), were unmarried (89.73%), earned a monthly income of less than 4000 CNY (60.08%), and were living with relatives (63.31%). The different mean scores of turnover intention corresponding to various socio-demographic characteristics of novice nurses are shown in Table 1. Turnover intention differed significantly by monthly income (*P* < .05). High turnover intention was observed among novice nurses who did not live with relatives (*P* < .01).

### 3.2. Respondents’ turnover intention, professional identity, and MMSS

As presented in Table 2, turnover intention levels were high and above among 54.37% (286/526) of the novice nurses, and low and below among 45.63%. Among a total of 526 novice nurses, professional identity levels were disapproval in 4.18%), general identity in 76.05%), and high identity only in 19.77%). The MMSS scores of novice nurses were investigated, and the results revealed that 52.09% were neutral (neither satisfied nor dissatisfied), 19.77% were dissatisfied, and 28.13% were satisfied. The mean scores for turnover intention, professional identity, and MMSS were 13.02 ± 3.94, 36.17 ± 7.98, and 111.02 ± 21.46, respectively. The average score of the dimension “Possibility of gaining an external job” showed that the three dimensions of turnover intention were the highest among novice nurses. Among the eight subscales of the MMSS, the average score of items corresponding to “Relations with colleagues” was the highest, while the average score of that corresponding to “Benefits” was the lowest among novice nurses.

**Table 2:** Turnover intention, professional identity, McCoskey/Mueller satisfaction scale levels of novice nurses (N = 526).

Dimensions	N (%)	Mean	SD
Turnover intention		13.02	3.94
Very low	47 (8.94)		
Low	193 (36.69)		
High	263 (50.00)		
Very high	23 (4.37)		
Professional identity		36.17	7.98
Disapproval	22 (4.18)		
General identity	400 (76.05)		
High identity	104 (19.77)		
McCoskey/Mueller satisfaction scale		111.02	21.46
Very dissatisfied	3 (0.57)		
Moderately dissatisfied	101 (19.20)		
Neutral (neither satisfied nor dissatisfied)	274 (52.09)		
Moderately satisfied	112 (21.29)		
Very satisfied	36 (6.84)		

SD = standard deviation

### 3.3. Bivariate correlations between turnover intention, professional identity, and MMSS

The results of the Pearson’s correlation analysis are shown in Table 3. Both professional identity (*r* = −0.459) and MMSS (*r* = −0.517) showed a significant moderate negative correlation with turnover intention (*P* < .01). In addition, a strong positive association was found between professional identity and MMSS (*r* = 0.723, *P < *.01).

**Table 3:** Correlations between turnover intention, professional identity, and MMSS (N = 526).

Variables	1	2	3	4	5	6	7	8	9	10	11	12	13	14
Turnover intention														
1.Turnover intention general	–													
2.Possibility of leaving	0.897**	–												
3.Motivation to find other jobs	0.896**	0.794**	–											
4.Possibility of obtaining external work	0.749**	0.475**	0.463**	–										
5.Professional identity general	−0.459**	−0.509**	−0.423**	−0.228**	–									
**MMSS**														
6. MMSS general	−0.517**	−0.540**	−0.476**	−0.293**	0.723**	–								
7.Benefits	−0.487**	−0.487**	−0.427**	−0.322**	0.662**	0.879**	–							
8.Scheduling	−0.495**	−0.527**	−0.447**	−0.279**	0.693**	0.943**	0.850**	–						
9.Balance between family and work	−0.483**	−0.499**	−0.433**	−0.292**	0.677**	0.915**	0.864**	0.879**	–					
10.Relations with colleagues	−0.434**	−0.453**	−0.420**	−0.222**	0.593**	0.845**	0.663**	0.766**	0.731**	–				
11.Social opportunities	−0.477**	−0.504**	−0.432**	−0.272**	0.680**	0.930**	0.773**	0.819**	0.813**	0.757**	–			
12.Opportunities for professional development	−0.468**	−0.491**	−0.430**	−0.262**	0.682**	0.940**	0.780**	0.835**	0.820**	0.742**	0.920**	–		
13.Praise and recognition of work	−0.458**	−0.484**	−0.438**	−0.236**	0.642**	0.932**	0.740**	0.831**	0.799**	0.819**	0.862**	0.892**	–	
14.Control and responsibility for work	−0.491**	−0.510**	−0.471**	−0.261**	0.671**	0.954**	0.772**	0.866**	0.820**	0.860**	0.880**	0.898**	0.922**	–

MMSS = McCoskey/Mueller satisfaction scale

** *P* < .01

### 3.4. Multivariate linear regression analysis

Table 4 presents the results of the multivariate linear regression analysis. Turnover intention was taken as the dependent variable, and “Whether living with relatives, “Professional identity,” and the eight dimensions of the MMSS were taken as independent variables. As shown in Table 4, the results showed that “Whether living with relatives,” “Professional identity,” “Control and responsibility for work,” and “Benefits” jointly accounted for 29.9% of the variance in novice nurse’s turnover intention (*F* = 56.892; *P* =.000; *R*^2^ = 0.304, Adjusted *R*^2^ = 0.299).

**Table 4:** Multiple linear regression analysis of turnover intention (N = 526).

Dependent variables	Independent variables	B (unstandardized coefficients)	β (standardized coefficients)	*t*	*P*
Turnover intention	Whether living with relatives	1.082	0.132	3.578	.000
	Professional identity	−0.087	−0.176	−3.389	.001
	Control and responsibility for work	−0.248	−0.214	−3.506	.000
	Benefits	−0.284	−0.186	−3.072	.002

## 4. Discussion

It is worth noting that the turnover intention of novice nurses was influenced by “Whether living with relatives” and “Monthly income,” which is comparable to previous research findings.^[42,46–48]^ A study involving 382 health workers in Ethiopia found that living away from family was an influencing factor with regard to turnover intention among health workers.^[46]^ This could be due to the high costs of living and lack of stability experienced by novice nurses.^[46]^ In addition, Zhao^[49]^ found that social support directly affected turnover intention, and support from relatives was identified as an important component of social support for novice nurses. Novice nurses who live with their relatives also enjoy better social support, which can reduce turnover intentions. Cao et al ^[48]^ investigated 12,291 Chinese nurses and found that high turnover intention was associated with low salaries. Zhang et al^[42]^ surveyed 3236 general practitioners and found that income level was associated with turnover intention. Low income made it harder to attract and retain novice nurses.

The mean score of turnover intention acquired in this study was 13.02, indicating high turnover intention among novice nurses in China. A total of 54.37% of novice nurses had a high or very high turnover intention, showing that more than half of them had considered leaving the nursing profession. These findings are similar to those of previous studies^[20,48,50–52]^ Furthermore, the level of turnover intention was higher in the current study compared with Gong’s research which surveyed 315 newly graduated nurses in Sichuan province, China (mean score of 12.24).^[21]^ In this study, the prevalence of turnover intention was 54.37% among novice nurses in China, which was higher than the rates of 44.40% reported in China,^[21]^ 23.01% observed among intensive care nurses in Iran,^[17]^ 6.90% found among intensive care nurses in Belgium,^[53]^ and 27.7% pooled sample of intensive care nurses from a meta-analysis.^[19]^ High turnover intention among novice nurses may be attributed to the fact that Chinese nurses are required to complete 2 years of standardized training after graduation, and the obligation to rotate different clinical departments in the training period. In this study, the majority of novice nurses had less than 2 years of work experience (69.20%). They tended to find role transitioning difficult and experienced intense physical, emotional, and mental confusion, as well as pressure, low salaries, and high levels of uncertainty, all of which contribute to high turnover intention.^[29,54,55]^

The current situation regarding the turnover intention of novice nurses is worthy of our attention. Novice nurses’ professional identity was at a medium level, indicating that their professional identity was not high. The level of professional identity in this study was lower than that observed in Ren et al’s^[56]^ study. The survey results revealed that the level of high identity of novice nurses was only 19.77%, whereas it was found to be 56.99% in Ren’s^[56]^ study involving 1009 nurses. Job satisfaction among novice nurses was moderate rather than very high. This finding was in agreement with those of previous studies, such as Wang, which investigated 560 nurses in Mainland China,^[35]^ and Almansour’s study involving 743 nurses in Saudi Arabia.^[37]^ In addition, the current study also found that “Relations with colleagues” scored the highest, while “Benefits” scored the lowest, which concurred with the results obtained by Wang.^[35]^

This study is the first to link professional identity and job satisfaction to turnover intention among novice nurses. This is striking since the results revealed that both professional identity and job satisfaction had a negative impact on turnover intention among novice nurses. The findings indicate that high turnover intention is associated with a poor sense of professional identity and low levels of job satisfaction among novice nurses. This finding is consistent with the results of Sabanciogullari and Dogan^[14]^ and Huet al^[26]^ among nurses. A similar finding was also reported by Zhang et al^[42]^ involving 3236 general practitioners, and Zhang et al^[39]^ study with 1244 village public health service providers. In the present study, the correlation between professional identity and turnover intention (*r* = −0.459) was stronger than that observed by Hu et al^[26]^ (*r* = −0.342) and Zhang et al^[39]^ (*r* = −0.09). Moreover, the correlation between job satisfaction and turnover intention (*r* = −0.517) was stronger than in Huet al^[26]^ study (*r* = −0.501) and Zhang et al^[39]^ study (*r* = −0.460). These conflicting results may be due to the use of different measurement tools.

The results of the multivariate linear regression analysis also showed that high turnover intention was associated with novice nurses who were “Whether living with relatives” and those who had a weak “Professional identity,” as well as low scores on subdomains of job satisfaction including “Control and responsibility for work” and “Benefits,” which jointly accounted for approximately 30% of the total variance in turnover intention among novice nurses. Given that many factors affect novice nurses’ turnover intention, this outcome is crucial. In this study, more than half of the novice nurses considered leaving the nursing profession, which was related to a weak sense of professional identity and job satisfaction. Novice nurses may have considered that the professional identity of nursing was a frequently misunderstood concept and that it was not commonly used in nursing practice, which made it more difficult to further refine their professional identity as novice nurses integrated into the nursing profession.^[27]^ Therefore, novice nurses were found to have a poor sense of professional identity, which affected the level of turnover intention. The latter is also influenced by work-related stress, high workloads, perceived lack of job control and responsibility, and poor benefits, which are a reality shock for novice nurses.^[28,57]^ A series of measures should be taken to promote a stronger sense of professional identity among novice nurses to weaken the influence of low job satisfaction on turnover intention. Philippa^[27]^ suggested that novice nurses can develop their professional values in nursing, nursing knowledge, and skills, so as to ensure that they are up-to-date with current practice. Moreover, they should actively engage in effective collaboration, which should enhance their professional identity. In addition, greater attention should be paid to the remuneration and benefits awarded to novice nurses to ensure fair and efficient compensation.^[58]^ Moreover, attention should also be paid to lifting the ability to “Control and responsibility for work” of novice nurses, which can help to improve job satisfaction among this group, thereby reducing turnover intention.

This study had a few limitations that should be considered. First, this study utilized a cross-sectional design, which means that caution should be exercised when attempting to infer a causal relationship between turnover intention, professional identity, and job satisfaction. Second, the variables in this research were investigated based on self-report measures; therefore, response bias may have had an impact on the results. Third, convenience sampling may have affected the representativeness of the sample. Therefore, the results cannot be generalized to all novice nurses in China. Despite these limitations, the current study is significant because it conducted a broad investigation of the association between turnover intention, professional identity, and job satisfaction among novice nurses.

## 5. Conclusions

This study was the first to examine the association between turnover intention, professional identity, and job satisfaction among novice nurses. In this study, high turnover intention was found among novice nurses, of whom 54.37% had a high or very high turnover intention. Levels of professional identity and job satisfaction were moderate, rather than high. “Whether living with relatives” and “Monthly income” affected turnover intention among novice nurses. A noteworthy finding is that both professional identity and job satisfaction had a negative impact on turnover intention among novice nurses. Furthermore, this study also found that certain independent variables, namely “Whether living with relatives,” “Professional identity,” “Control and responsibility for work,” and “Benefits” were highly predictive of turnover intention levels among novice nurses. Hence, these potential predictors of turnover intention should be considered, and intervention research should be implemented to reduce turnover intention among novice nurses.

## Acknowledgments

The authors acknowledge the cooperation of the nurses who participated in the study.

## Author contributions

**Conceptualization:** Ying Zhong, Chang-ju Liao.

**Data curation:** Ying Zhong, Huan Ma, Cui-Cui Zhang, Qin-Ying Jiang, Jun Li, Yu-Fen Liang, Li Shu.

**Formal analysis:** Ying Zhong, Huan Ma.

**Investigation:** Ying Zhong, Huan Ma, Cui-Cui Zhang, Qin-Ying Jiang, Jun Li, Yu-Fen Liang, Li Shu.

**Methodology:** Chang-ju Liao.

**Project administration:** Chang-ju Liao.

**Software:** Ying Zhong, Huan Ma, Cui-Cui Zhang, Qin-Ying Jiang.

**Supervision:** Chang-ju Liao.

**Writing - original draft:** Ying Zhong, Huan Ma, Cui-Cui Zhang, Chang-ju Liao.

**Writing - review & editing:** Ying Zhong, Huan Ma, Cui-Cui Zhang.
